# Supplementary material for: Learning curve and surgical outcome of robotic assisted colorectal surgery with ERAS program
Source: Sci Rep. 2022 Nov 29;12:20566. doi: 10.1038/s41598-022-24665-w (PMC9709162; doi:10.1038/s41598-022-24665-w)
Supplement: Supplementary file 1 — Supplementary Information 1. [file 41598_2022_24665_MOESM1_ESM.zip › LSC Raw data-Ñ[▒K/086/N_ñw╝╨░O▒Kñσ.pdf]

W83 / W83

臺中榮民總醫院
手術室護理記錄

[redacted]

基本資料

- 室別： 二醫手術室-31室，手術科別： CRS，手術類別： 常規，日期： 2020/07/22
- 進入時間： 1150 ； 麻醉開始時間： 1150 ； Time Out時間： 1250 ； 劃刀時間： 1251 ； 手術結束時間： 1600 送出時間： 1615 ；
- 麻醉方式： GE

  診斷：s/p loop T with vulvulus.

  手術名稱：Laparoscopic Anterior resection and anastomosis-benign

  術前預防性抗生素：
    - 抗生素名稱 ： Veterin 1GM IVD
    - 時間： 1237
    - 上次簽章: 葉新娟

  ，追加預防性抗生素：
- 手術醫師： 林俊余，楊曙榮，陳凱祥，吳柏寬
- 麻醉醫師： VS 徐煥
-
    - 刷手護士A ： 林宜旻，時間： 1150，接替刷手一： 吳雅華，時間： 1230-1300，接替刷手二： 林宜旻，時間： 130-1500
    - 流動護士A ： 吳雅華，時間： 1150，接替流動一： 葉新娟，時間： 1200-1300，接替流動二： 吳雅華，時間： 1300-1500
- 更多接替及流動護士： 接替刷手三： 陳昱穎，時間： 1500-1615，接替流動三： 吳若綺，時間： 1500-1615

皮膚狀況

A-V Shunt： 無

術前： 完整

記錄者： 上次簽章: 葉新娟

術後： 不完整

- 7. 壓紅不可白

部位(術後)：

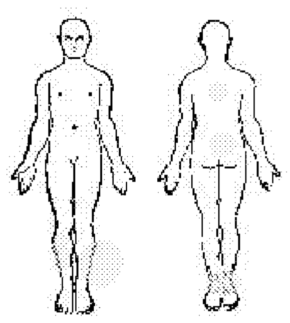

1.左足踝1*1cm壓紅不可白
2.背部脊椎4*2cm壓紅不可白
3.薦骨5*4cm壓紅不可白
4.雙足跟3*3cm壓紅不可白
5.雙側脛骨10*1cm壓紅不可白
6.鼻頭1*1cm壓紅不可白

記錄者： 吳若綺

手術準備

手術臥式 單一手術臥式

-
    - 手術臥式： 截石臥式
    - 使用附件： 矽膠枕頭，腳架

- ○ 皮膚準備： 10%Aq Betadine , Hibiscrub , 2% Chlorhexidine
- ○ 電燒： 使用電燒: 電極片位置： 其它： 迴路電極墊 , 記錄者： 上次簽章: 葉新娟

止血帶T： 無

植入物

無

標本送檢

- 細菌培養 無
- 細胞學 無
- 冰凍切片 無
- 病理標本
  組織名稱一：T-D colon
- 截肢 無

標本交付 標本： 壹 袋 , 標本： 零 盒 , 標本： 零 瓶 , 細胞學： 零 管 , Culture Tube： 零 管 , 被交付醫師： CR 楊曙榮 ,

- 記錄者： 吳若綺
- 時間： 1610

傷口術後敷料

傷口敷料： 傷口敷料： 無

- 請於圖上標示部位：

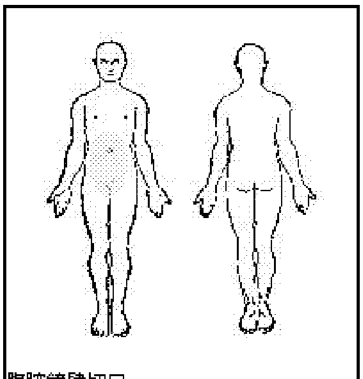

- 傷口敷料：MEPORE

導管

導管	天數	導管資訊	插入日期	插入時間	更換日期	拔除日期	拔除時間	拔除原因	預計拔除/更換	預計拔除/更換日期
Foley(1)	1	號碼:16 Fr.; 部位:膀胱;	2020/07/22	1230						
Sump NG Tube (1)	1	號碼:16 Fr.; 部位:胃; 註記:Fix 55cm;	2020/07/22	1240						
A-line(1)	1	號碼:20 G; 部位:前臂;	2020/07/22	1245						
Drainage Catheter (PCN)(1)	43	深度:21 cm-->13cm; 部位:腎臟; 註記:R'T;	2020/06/10	1200						
Drainage Catheter (PCN)(2)	43	深度:12 cm-->11cm; 部位:腎臟; 註記:L'T;	2020/06/10	1201						

用物計數

- 手術中出血量： Minimal c.c.
- 手術中尿量： 250 c.c.
-
  - ○ 其它： 無

用物計數： 多切口同時縫合

- 1.器械： 正確 , 2.縫針： 正確 , 3.刀片： 正確 , 4.紗布： 正確 , 5.腹部墊： 未使用 , 6.紗球： 未使用 , 7.PEANUTS ： 未使用 , 8.紗條： 未使用 , 9.絲帶： 未使用 , 10.針頭： 未使用 , 11.Vessel loop： 未使用 ,

12.Boots： 未使用，13.Jelco： 未使用，14.棉條： 未使用，

- 第一次：
  - 刷手護士 ： 陳昱穎
  - 流動護士： 吳若綺
  - 時間： 1500

,

- 第二次：
  - 刷手護士 ： 陳昱穎
  - 流動護士： 吳若綺
  - 時間： 1525

,

- 第三次：
  - 刷手護士 ： 陳昱穎
  - 流動護士： 吳若綺
  - 時間： 1545

備註

備註：NIL

- 記錄者： 吳若綺
- 時間： 1615

填寫護理人員 吳若綺

正式摘要

W83 / W83

doctorRank:B
